# Goals of Care Discussions in Medical Training: Integrating Palliative Care for Holistic, Patient-Centered Care

**DOI:** 10.3390/healthcare14091222

**Published:** 2026-05-01

**Authors:** Celine Rochon, Farzana Hoque

**Affiliations:** 1 Saint Louis University School of Medicine, St. Louis, MO 63104-1016, USA; celine.rochon@health.slu.edu; 2 Department of Medicine, Saint Louis University School of Medicine, St. Louis, MO 63104-1016, USA

**Keywords:** goals of care discussions, palliative care training, serious illness communication, communication frameworks, patient-centered care, hospital medicine, artificial intelligence in healthcare

## Abstract

**Background:** Goals of care discussions are essential communication skills in medical training that bridge patient values with clinical decision-making. Integrating palliative care principles into these conversations enables holistic, patient-centered care, yet medical trainees often lack structured preparation for these critical interactions. **Objective:** This narrative review examines how medical training can effectively integrate palliative care approaches into goals of care discussions through structured communication frameworks, interdisciplinary collaboration, and emerging innovations to promote patient-centered outcomes. **Methods:** This narrative review is conducted using a structured literature search that includes relevant studies pertaining to goals of care (GOC) discussions, evidence-based communication frameworks, and communication training curricula. Databases used were PubMed and Google Scholar, using articles published between 2000 and 2025. The following keywords were used in our search: “SPIKES”, “REMAP”, “SUPER”, “serious illness conversation”, “goals of care,” “end of life,” “holistic care,” “palliative care,” and “medical education.” Exclusion criteria were used to select those relevant to inpatient care and training in inpatient settings. Studies in an outpatient setting were excluded. Findings were reviewed and synthesized to identify types of training approaches. An emphasis on clinical outcomes including patient satisfaction, hospice utilization, ICU transfers, and intervention intensity were examined. Educational barriers and facilitators—including communication training curricula, cultural competency, language considerations, and multidisciplinary team involvement—were evaluated. Emerging technologies supporting clinician education and practice were also assessed. **Results:** Training in structured communication frameworks improves patient–physician relationships, reduces patient anxiety, and increases family satisfaction. Early palliative care integration through effective discussions leads to increased hospice awareness and utilization while reducing burdensome interventions. Key educational facilitators include dedicated communication skills training, multidisciplinary team participation (including chaplains and palliative care specialists), and AI-assisted documentation tools that support learning while preserving humanistic clinician–patient interactions. **Conclusions:** Integrating palliative care principles into medical training for goals of care discussions is essential for developing patient-centered clinicians. Combining structured communication frameworks, interprofessional education, targeted skills training, and technological support creates a comprehensive educational approach that prepares trainees to elicit patient goals, create individualized care plans, and deliver holistic care that honors patient values.

## 1. A Real Case

A 78-year-old man with no known medical history, who had not seen a physician in more than 40 years and lived alone without any family support, was admitted with abdominal pain, nausea, and vomiting. CT imaging revealed a strangulated inguinal hernia requiring urgent surgical intervention, along with an incidental finding of a widely metastatic disease. Although the surgical team recommended emergency repair to prevent bowel necrosis and sepsis, the patient—stunned by the unexpected cancer diagnosis—declined surgery and asked to be discharged, refusing any further diagnostic testing. As the hospitalist, you are now faced with initiating an urgent goals of care discussion with a patient who has intact decision-making capacity but no surrogate, a newly identified widely metastatic illness, and a life-threatening condition requiring time-sensitive intervention.

## 2. Introduction

Goals of care discussions provide a structured approach to patient-centered medical care by prioritizing a patient’s values and objectives, which in turn guide decisions about therapeutic interventions [1]. The term is most often applied in the context of serious illness, typically when prognosis is poor or death may be imminent [1]. These conversations not only support individualized medical decision-making, but also strengthen the therapeutic alliance between patients and clinicians and reduce non-beneficial treatments [2]. Despite their value, goals of care discussions in the hospital setting are often suboptimal or underutilized. Common contributing factors include lack of clinician confidence, uncertainty about who is responsible for initiating the conversation, limited training, inadequate documentation systems, and poorly timed discussions [1]. In one study, only 36.4% of eligible hospitalized patients had a documented goals of care conversation [3]. Cost implications also warrant attention, as healthcare expenditures, a significant portion being end-of-life care, increase when patients receive unwanted or aggressive treatment [4,5]. Such actions may be avoided through effective advance care planning and goals of care conversations.

Within palliative care, GOC discussions function as a means for holistic assessment, which evaluates a patient’s well-being in a broad, multidimensional context. A perspective within palliative care is that the most effective version should not neglect the lived experiences of a patient, instead adopting a holistic and biopsychosocial model rather than a purely biomedical one [6]. The National Consensus Project (NCP) Clinical Practice Guidelines for Quality Palliative Care outlines that comprehensive assessment goes beyond considering only physical symptoms, and factors in emotional, psychological, ethical, cultural or spiritual influences [7]. Through GOC discussions, clinicians create opportunities for patients to express their concerns and understanding of their illness and treatment, operationalizing a holistic approach through intentional inquiry.

When exploring how holistic assessment can be performed, it is important to consider that communication frameworks give structure and guidance during serious illness discussions. Frameworks used to develop communication skills and navigate emotionally complex conversations in the setting of advanced illnesses include SPIKES, REMAP, and the Serious Illness Conversation Guide. Existing literature widely reports a lack of clinician confidence and ability to carry GOC conversations, bridged by frameworks that aim to improve communication outcomes. However, there is a large focus on how these frameworks operate in controlled or specialty-specific settings and limited synthesis on how these tools are taught and developed in early training and maintained across different stages of learning. Standardizing the approach to goals of care discussions may help address not only implications of healthcare utilization and costs, but also improve consistency and quality in patient-centered decision-making that strengthens the approach to holistic assessment.

This narrative review aims to critically evaluate communication frameworks and propose system-level strategies to counteract barriers to implementation, particularly in hospital-based care.

## 3. Communication Frameworks

**Figure 1:**
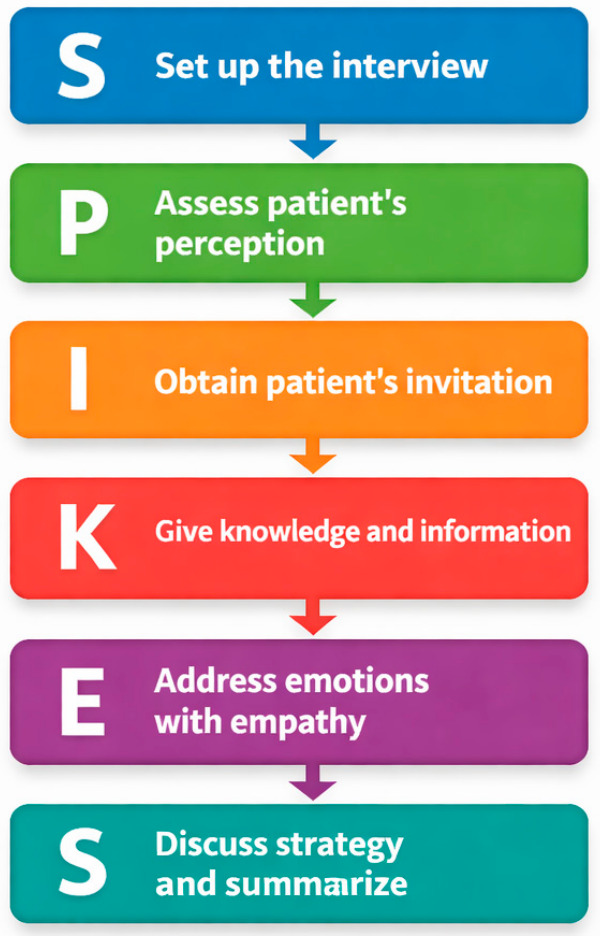
SPIKES framework: A six-step approach commonly used to guide clinicians when delivering serious medical information [8].

Hospitalists frequently manage high clinical workloads and complex decision-making under time pressure, which can limit the opportunity for thoughtful goals of care discussions and holistic assessment. The use of structured communication frameworks provides an evidence-based method to conduct these conversations more efficiently and consistently, supporting clinician confidence, reducing cognitive burden, and ensuring that patient values remain central even in fast-paced inpatient environments. One widely taught communication framework is the SPIKES protocol, a six-step model that addresses setting, perception, invitation, knowledge, emotions, and strategy (Figure 1) [8]. This approach creates a safe environment, allows assessment of the patient’s understanding through open-ended questions, provides information with clarity and transparency, responds empathetically to emotional reactions, and remains future-oriented when formulating a therapeutic plan [8].

**Figure 2:**
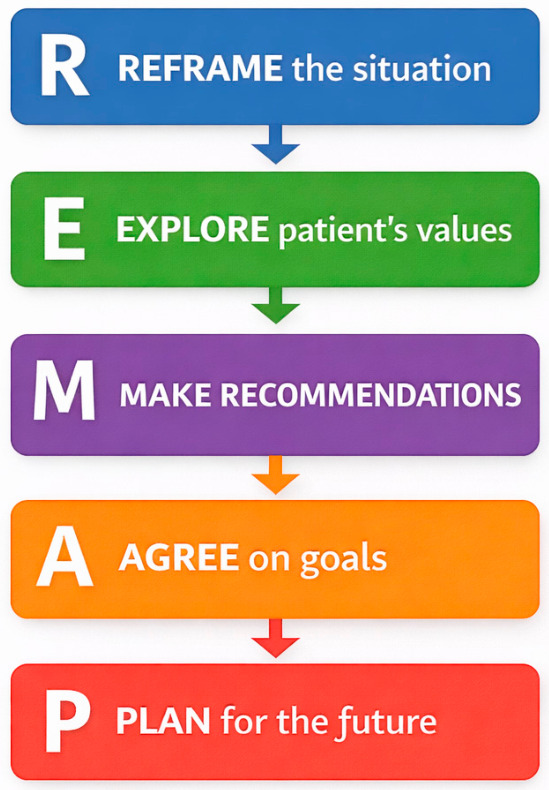
REMAP framework: Key steps used to structure goals of care discussions and align treatment decision with patient values [9].

**Figure 3:**
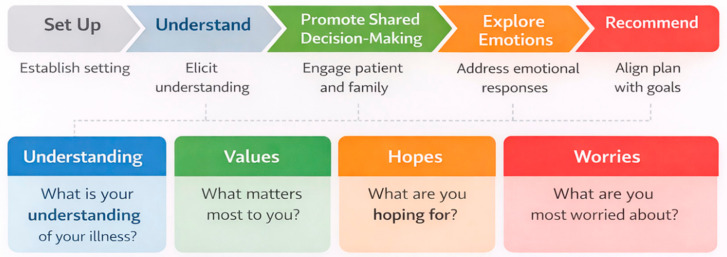
Integration of SUPER framework and the Serious Illness Conversation Guide: A summary of core domains used to facilitate conversations with patients facing serious illness [10,11].

Another communication framework, known as REMAP, supports shared decision-making and discussions about end-of-life care. This model takes a similar structured approach to goals of care conversations by emphasizing five key steps: reframing the medical situation, expecting and validating emotion, mapping out patient values and goals, aligning the clinical team with those goals, and proposing a care plan that reflects them (Figure 2) [9]. In the presented case of a patient with newly diagnosed metastatic disease refusing surgical intervention, frameworks such as REMAP are designed to reframe the medical situation and investigate the patient’s values, prioritizing their goals rather than advocating an urgent and unwanted surgery. SUPER is another mnemonic used to explore patient emotions and comprehension, organizing conversations by first setting them up, understanding prognosis and priorities, anticipating emotions, and conducting a final review of a therapeutic plan (Figure 3) [10]. The Serious Illness Conversation Guide is utilized in ICU settings, a tool designed to prompt sensitive questions and explore patients’ fears, values, and worries (Figure 3) [11]. Together, these frameworks show a process of initiating a conversation and ways to delve into deeper inquiry.

Collectively, these frameworks help clinicians navigate complex discussions in the inpatient setting by centering the conversation on patient priorities, therapeutic goals, and value-based decision-making; however, their effectiveness can vary depending on clinical context. SPIKES is widely used by oncologists, especially when having to deliver serious news. The questions asked may be limited in their ability to explore longitudinal decision-making, which REMAP and the Serious Illness Conversation Guide may be more suited for. Due to the innate seriousness of end-of-life care, these conversations may not be ideally executed in one sitting, and require multiple conversations. Though the Serious Illness Conversation Guide offers more in-depth exploration, it may not perform best in fast-paced, time-constrained settings. Step-wise approaches can be dependent on a patients’ willingness to engage, presenting a risk that a conversation might end prematurely. Communication frameworks outline an approach to difficult conversations, but results can still depend on one’s level of expertise and clinical judgment. Medical students may benefit from a structured, step-wise communication that SUPER provides. Higher learners such as residents and fellows are a greater targeted and evaluated group when studying effective implementation, since their clinical experience allows them to better engage with REMAP and the Serious Illness Conversation Guide. Introducing simplified frameworks may help students build foundational communication skills as their medical knowledge naturally grows and informs decision-making abilities. Understanding the applicability and reproducibility of frameworks across different healthcare settings remains important for effective integration and investment in training.

## 4. Impact on Clinical Outcomes

Effective goals of care discussions are associated with multiple positive clinical and experiential outcomes. A summary of key studies evaluating goals of care communication, including study design and strength of evidence, is provided in Table S1. Patients who engage in GOC conversations demonstrated improved emotional well-being and higher family satisfaction [12]. These discussions also promote shared decision-making by actively involving patients—and family members when appropriate—in clarifying their values, priorities, and goals, thereby fostering meaningful patient engagement rather than them being passive recipients of medical decisions [13]. Because every patient has unique priorities and definitions of an acceptable quality of life, an individualized approach can align with what matters most to the patient. In addition, palliative care consultation specifically for goals of care discussions has been linked to significantly higher rates of hospice enrollment at the time of hospital discharge compared with patients who did not receive such consultation [14]. Although these conversations often occur during advanced stages of illness, evidence demonstrates that they can reduce admission to the intensive care unit, limit the use of mechanical ventilation, and decrease other aggressive interventions such as cardiopulmonary resuscitation [15]. Without goals of care conversations, clinical outcomes may become suboptimal with respect to the medical and emotional distress of a patient.

Though hospice utilization increases and ICU admission decreases, these outcomes may be influenced by the availability of resources which is inconsistent across healthcare systems. Strength of evidence varies across study designs, with possible confounding factors influencing non-randomized and observational studies, such as disease severity and patient selection bias. The Serious Illness Care Program randomized trial prompts the question of generalizability, since improvements in communication were seen with groups of advanced cancer patients, though it is more ambiguous whether these findings would translate to other serious illness groups, such as chronic obstructive pulmonary disease and congestive heart failure patients, where illness trajectory may be more unpredictable. These limitations highlight the need for studies beyond oncology to gain insights into heterogeneous patient populations and real-world clinical settings.

## 5. Barriers

Although goals of care discussions are highly valuable, several barriers stemming from psychological, social, and cultural categories may limit their effectiveness and consistent implementation in clinical practice. This complexity is also illustrated in the clinical case, where a surrogate decision-maker was not present, and the patient’s emotions were extremely heightened. One contributing factor is patient fear, which can lead to misunderstanding of treatment options and impaired decision-making; addressing these concerns through validation and reassurance is an essential element of effective communication [12]. Religious or spiritual beliefs represent another commonly overlooked dimension, often due to clinicians’ discomfort, lack of training in discussing faith, or a tendency to prioritize medical expertise over a patient’s spiritual preference [16]. Language barriers may further impede communication, as cultural differences and the limitations of medical terminology in translation can contribute to confusion and mistrust [17]. Additional challenges include patient discomfort with prognostic uncertainty and the time constraints of inpatient care, which may limit clinicians’ ability to engage in thoughtful, unhurried conversations during daily rounds [18]. Goals of care discussions can also be especially difficult for older adults who experience sensory or cognitive impairments, such as hearing loss, visual disability, or dementia. Patients who lack decision-making capacity and have no available surrogates such as those who live alone or are estranged from family, incarcerated, or admitted with altered mental status create further ethical and logistical complexity. Moreover, clinicians may mistakenly defer these conversations with the belief that they can be addressed in the outpatient setting after discharge, which places medically fragile patients at risk for unwanted, aggressive treatment and potentially preventable suffering. Another common barrier is the misconception among patients and families that palliative care consultation means the medical team will “give up.” This misunderstanding may lead to resistance, even when palliative involvement is intended to enhance comfort, clarify values, and support concurrent treatment plans.

## 6. Educational and System-Level Strategies

Addressing these barriers requires targeted educational and system-level changes in order to establish communication as routine practice. For example, facilitation can be improved through the communication frameworks SPIKES, REMAP, SUPER, and the Serious Illness Conversation Guide. Additional interventions include decision-support tools, clinician training programs, multidisciplinary collaboration, and the involvement of spiritual care teams. Workflow can be supported by predictive models—such as mortality risk scores—that help identify key clinical moments when goals of care discussions should occur [19].

Developing competent communication skills must begin early in medical training [20]. Educational approaches such as role-playing, simulation, and standardized patient encounters allow learners to practice difficult conversations in a safe environment, identify gaps in interpersonal skills, and refine communication techniques without risk to patients [20,21]. Simulation-based scenarios are consistently rated by residents as effective for building confidence, receiving streamlined faculty feedback, and encouraging self-reflection [22,23]. Communication training programs such as Oncotalk have been utilized by fellows, a workshop tailored to discussing transitions to palliative care in the setting of cancer progression [24]. Empathy is a critical component of goals of care conversations, and teaching it requires intentional instruction—not assumption [22]. Medical students can develop empathic competencies through facilitated debriefing, reflective writing, small-group discussions, and supervised encounters with simulated or real patients [21]. Despite evidence of training modalities, implementation in real-world curricula remains inconsistent and not standardized across U.S. medical schools [25], highlighting the need for educational reform that overcomes competing curriculum demands and limited resources, ultimately optimizing exposure to goals of care training.

Palliative care assessment is multi-faceted; thus, collaboration amongst an interdisciplinary team (IDT) is important as a system-level strategy to implement holistic goals of care discussions. An IDT can perform mental status screenings, a process supplemented by the roles of social workers who further evaluate mental health [7]. For religious patients, chaplains play an essential role in addressing existential distress and supporting patients and families during serious illness [26]. For patients lacking decision-making capacity, surrogate decision-makers play an important role in maintaining patient outcomes [27]. Clinicians should work closely and actively converse with surrogates to clarify underlying values that impact judgment and goal-concordant care. Empathy is also a critical component of goals of care discussions, facilitating meaningful patient engagement and strengthening alignment between clinical decision-making and patient values [22]. Ensuring that patients, families, and clinical teams share an understanding of the care plan creates a unified agenda and promotes treatment that reflects patient-centered goals.

## 7. Future Directions

Emerging technologies are seen as supportive features in educational and clinical training. Artificial intelligence (AI) has emerged as a novel adjunct to traditional communication methods [28]. Goals of care discussions can now be supported by AI platforms that help overcome language barriers, generate empathic phrasing, or synthesize complex medical information while preserving the humanistic role of the clinician [29]. Natural language processing (NLP), a form of AI that extracts meaning from written text, has been used to detect and classify goals of care documentation in electronic medical records (EMR), improving the ability to track conversations across care settings, evaluate intervention outcomes, and identify whether discussions have occurred prior to hospitalization [28,29,30]. Integrating AI into clinical practice is still used in caution due to ethical concerns and patient privacy. Due to its ongoing refinement, areas of limitations include medical accuracy, which can be harmful for providers and patients if incorrect information is generated. Over-reliance could diminish the presence of authentic physician and patient interaction, or systemic errors and inequities could impact vulnerable populations by unintentionally treating groups differently. This further shows that AI is a supplemental tool, needing further research to better ensure accuracy and protection of sensitive information.

## 8. Conclusions

Goals of care discussions are essential in hospital medicine, where clinical conditions can deteriorate quickly. Pertinent themes to recognize in the initial case are that decisions must often be made under time pressure, making alignment with patient values critical, especially when proposed medical interventions are not desired. Instrumentally, goals of care conversations operate as a central mechanism for executing holistic palliative care. Structured frameworks such as SPIKES, REMAP, SUPER and the Serious Illness Conversation Guide help clinicians navigate these discussions by exploring what might be affecting a patient emotionally and psychosocially. However, the effectiveness of these frameworks depends on clinician training and context, and incorporation into workflow. While efforts grow to improve GOC training, a focus on embedding learning throughout medical education can improve residents when they become frontline physicians. This may also include developing efficient documentation systems, and greater emphasis on interdisciplinary collaboration. Emerging tools such as artificial intelligence and natural language processing may enhance documentation and timing but should serve as adjuncts—not substitutes—for empathetic, patient-centered communication. Ultimately, strengthening the consistency and quality of goals of care discussions in the inpatient setting is therefore not only an essential clinical skill, but an ethical responsibility and system-level priority. Integrating structured communication and holistic assessment ensures that hospitalization does not default to aggressive care by assumption, reflects what matters most to patients, and investigates the emotional, cultural, and social factors that shape their care preferences.

## Data Availability

No new data were created or analyzed in this study. Data sharing is not applicable to this article.

## Supplementary Materials

The following supporting information can be downloaded at: https://www.mdpi.com/article/10.3390/healthcare14091222/s1, Table S1: Summary of Key Studies Evaluating Goals of Care Communication and Clinical Outcomes.
