# Supplementary material for: Goals of Care Discussions in Medical Training: Integrating Palliative Care for Holistic, Patient-Centered Care
Source: Healthcare (Basel). 2026 May 1;14(9):1222. doi: 10.3390/healthcare14091222 (PMC13163983; doi:10.3390/healthcare14091222)
Supplement: Supplementary file 1 [file healthcare-14-01222-s001.zip › healthcare-4226854-supplementary.pdf]

Table S1. Summary of Key Studies Evaluating Goals-of-Care Communication and Clinical Outcomes

Study	Study Design	Population	Focus	Key Findings	Strength of Evidence
Bernacki et al. [11]	Randomized Controlled Trial	Advanced cancer patients	Serious Illness Care Program	Improved communication quality and earlier GOC discussions	Strong (RCT), but limited to oncology
Wright et al. [15]	Prospective Cohort	Advanced cancer patients	End-of-life discussions	↓ ICU use, ↓ aggressive care, improved caregiver outcomes	Moderate (observational), possible confounding
Starr et al. [14]	Propensity Matched Cohort	Hospitalized patients	GOC consultation	↑ hospice enrollment at discharge	Moderate, adjusted analysis but residual bias
Uyeda et al. [3]	Retrospective Cohort	Hospitalized and chronic illness	GOC documentation	Only 36.4% had documented discussions	Real-world data, no causality
Pollak et al. [10]	Pilot Study	Hospitalists	Communication coaching	Improved GOC quality and patient outcomes	Small sample size
Nagpal et al. [20]	Educational Intervention	Residents	Simulation training	↑ confidence in GOC discussions	Self-reported outcomes
Osgood et al. [22]	Educational Pilot Study	Neurology residents	Simulation training	Improved communication comfort	Limited generalizability

Schell et al. [19]	Implementation Study	Hospital systems	Mortality prediction model + GOC documentation	↑ GOC documentation rates	Observational, system-level variability
